# [Retracted] Suppression of the death gene BIK is a critical factor for resistance to tamoxifen in MCF-7 breast cancer cells

**DOI:** 10.3892/ijo.2025.5842

**Published:** 2025-12-23

**Authors:** Rubí Viedma-Rodriguez, Luis Arturo Baiza- Gutman, Alejandro García-Carrancá, Leticia Moreno-Fierros, Fabio Salamanca-Gómez, Diego Arenas-Aranda

Int J Oncol 43: 1777-1786, 2013; DOI: 10.3892/ijo.2013.2127

Following the publication of the above paper, a concerned reader drew to the Editor's attention that, in Fig. 6, the same data appeared to have been included in three of the flow cytometric plots featured in this figure (for the Non-treated, Scrambled and SiRNAi BIK experiments). Moreover, the control β-actin western blots in Figs. 2B, 4A and 8B appeared to be the same (although, in these cases, the lanes for the blots were labelled up identically, and so these apparent duplications may not have represented an incorrect assembly of these figures); similarly, the control blots in Fig. 7A and B were also matching. However, the control β-actin western blots in three other figure parts (Fig. 1C and 7A/B) were also duplicated, and in this case, the numbers of lanes in the gel slices were different / the lanes were labelled differently.

The authors were contacted by the Editorial Office to offer an explanation for these potential anomalies in the presentation of the data in this paper, although up to this time, no response from them has been forthcoming. Owing to the fact that the Editorial Office has been made aware of potential issues surrounding the scientific integrity of this paper, the Editor of *International Journal of Oncology* has decided that this paper should be retracted from the Journal on account of a lack of confidence in the presented data. The Editor apologizes to the readership for any inconvenience caused.

